# Thyroglobulin levels in COVID-19-positive patients: Correlations with thyroid function tests, inflammatory markers, and glucocorticoid use

**DOI:** 10.3389/fendo.2022.1031188

**Published:** 2023-03-09

**Authors:** Renata Świątkowska-Stodulska, Agata Berlińska, Ewelina Puchalska-Reglińska

**Affiliations:** ^1^ Department of Endocrinology and Internal Medicine, Faculty of Medicine, Medical University of Gdańsk, Gdańsk, Poland; ^2^ Dialysis Unit, 7 Navy Hospital, Gdańsk, Poland

**Keywords:** COVID-19, SARS-CoV-2, thyroid, thyroglobulin, TSH, glucocorticoids, IL-6, CRP

## Abstract

COVID-19 often results in generalized inflammation and affects various organs and systems. Endocrine research focused on the possible sequelae of COVID-19, with special interest given to the thyroid gland. Clinical problems such as thyroid function in non-thyroidal illness (NTI), autoimmune thyroiditis, and COVID-19-related subacute thyroiditis (SAT) quickly gained wide coverage. Thyrotoxicosis of various origins leads to the release of peripheral thyroid hormones and thyroglobulin (TG), the main glycoprotein contained within the thyroid follicular lumen. In our study, we evaluated TG levels in COVID-19-positive patients and investigated the possible relationships between TG, thyroid function tests (TFTs), and inflammatory markers. Our approach included separate subanalyses of patients who received and those who did not receive glucocorticoids (GCs). In the entire population studied, the concentration of TG tended to decrease with time (p<0.001; p1,2 = 0.025, p1,3 = 0.001, p2,3 = 0.003), and this pattern was especially clear among patients treated with GCs (p<0.001; p1,2=<0.001; p1,3=<0.001; p 2,3=<0.001). The concentration of TG differed significantly between patients treated and those not treated with GC at the second and third time points of observation (p=0.033 and p=0.001, consecutively). TG concentration did not differ between the patients with normal and abnormal TFTs. The correlations between TG, TFTs, and inflammatory markers were very limited. 19 patients had elevated TG levels, but a TFT pattern suggestive of thyrotoxicosis was not common in this group. There were no statistically significant differences between patients who met and those who did not meet the predefined combined primary endpoint.

## Introduction

After nearly three years of the pandemic, COVID-19 remains an important and widespread clinical problem. Multiple aspects of the disease were extensively studied, with the thyroid function in infected patients being one of the most interesting and complex topics in endocrinology. Clinical problems such as thyroid function in non-thyroidal illness (NTI), autoimmune thyroiditis, and subacute thyroiditis (SAT) related to COVID-19 gained wide coverage (1). The majority of thyroid function abnormalities appear mild and transient, often fading once the patients recover from COVID-19 (2, 3). The novel onset of thyroid pathologies was hypothesized to be due to the direct and indirect effects of SARS-CoV-2 on the gland; the direct effect could be tied to viral infiltration of thyroid tissue, while the indirect effect was due to the generalized and uninhibited release of cytokines (1, 4, 5).

Trending topics related to thyroid and COVID-19 included, among others, NTI, thyrotoxicosis, and SAT. It seems that especially SAT associated with COVID-19, after being reported for the first time in 2020 by Brancatella et al., quickly gained special interest (6). SAT is known to follow viral infections of various origins, therefore, its connection with COVID-19 caused by SARS-CoV-2 coronavirus is highly plausible (7, 8). However, original studies with larger numbers of participants remain scarce and most of the available literature consists of case reports, case series, or systematic reviews (9–12). At the same time, a growing body of data supports the idea of thyrotoxicosis triggered by COVID-19, most of which seems to be caused by uncontrolled immune activation (13, 14). In our article, the objective was to evaluate thyroglobulin (TG) levels in patients hospitalized with COVID-19, perceiving TG as a biomarker of thyroid damage. We correlated TG concentrations measured at different time points of follow-up with the results of thyroid function tests (TFTs) and inflammatory markers. If TG levels increased, we provided an in-depth analysis for individual cases. Finally, we compared TG concentrations between patients who met the predefined endpoints and those who did not. Whenever possible, the analyzes were conducted separately for the patients who received and those who did not receive GCs.

## Materials and methods

In our observational study, we analyzed 174 patients hospitalized for COVID-19. All recruited patients were adults (≥18 years) and COVID-19 infection was confirmed by a PCR test in all patients. Our project recruited new patients between 14 February 2021 and 1 December 2021 at the 7 Navy Hospital in Gdańsk, Poland.

The study was approved by the Independent Bioethics Committee for Scientific Research at the Medical University of Gdańsk, Gdańsk, Poland (permissions NKBBN/373/2020, NKBBN/373-96/2021, NKBBN/373-184/2021), and was conducted according to the Declaration of Helsinki. Patients provided their informed consent to participation; if patients were unable to consent in writing due to a poor general condition resulting from COVID-19 and/or other diseases, the recruiters made a clear remark on the consent card. The only exclusion criterion we implemented was the patient’s dissent to participate. The study was registered at ClinicalTrials.gov (NCT05070091).

Blood was collected for laboratory tests in the morning (6 a.m. - 8 a.m.) by qualified medical personnel. The collection took place at three preset hospitalization time points: on days 1, 4, and 10, later referred to as the first, second, and third time points, respectively. Biochemical analyzes were performed in contracted commercial laboratories, and detailed data on laboratory norms and methods are available as a **Supplementary Table 1**. The evaluated parameters included TFTs such as thyrotropin (TSH), total thyroxine (T4), free thyroxine (fT4), free triiodothyronine (fT3), reverse triiodothyronine (rT3), and TG, anti-thyroglobulin antibodies (anti-TG abs), C-reactive protein (CRP), interleukin-6 (IL-6), total blood count including leukocytes (LEU), neutrocytes (NEU), and lymphocytes (LYMPH). Demographic and anthropometric data were collected. Every day the blood was drawn, the patients’ basic vital parameters (heart rate, blood pressure, blood oxygen saturation) and supplementary oxygen demand were recorded. Data on concomitant diseases and medication use were collected (**Table 1**). 7 patients (4.0%) used GCs to treat their chronic conditions, and in these cases GCs were administered continuously after admission. Additional patients required GC treatment for COVID-19 – in total, 93 patients (53,4%) received GCs at least once during their hospital stay. Due to the use of GCs by some patients, the studied group was divided into two subgroups for statistical analysis: those treated with steroids (the glucocorticoid group – GCG) and those not treated with steroids (the no-glucocorticoid group - NGCG). GCG consisted of patients who received GCs at least once during their hospitalization and the medication was administered at least one day before blood collection.

**Table 1:** General characteristics at admission.

Parameter	Value
Mean age (years) ±SD	66.8 ± 15.1
Mean BMI (kg/m^2^) ±SD	27.6 ± 5.3
Female sex (number of cases/percentage* [%])	84/48.3
Supplemental oxygen use (number of cases/percentage* [%])	100/57.8
Death (number of cases/percentage* [%])	30/17.2
NIV/CPAP/HFNO (number of cases/percentage* [%])	12/6.9
Mechanical ventilation (number of cases/percentage* [%])	3/1.7
Catecholamine infusion (number of cases/percentage* [%])	6/3.5
Hospital stay for at least 10 days (number of cases/percentage* [%])	87/50
Steroid use at any point of observation (number of cases/percentage* [%])	93/53.4
Chronic steroid use before hospital stay (number of cases/percentage* [%])	7/4.0
Abnormal TFTs at any point of observation (number of cases/percentage* [%])	140/80.5
History of hypothyroidism (number of cases/percentage* [%])	18/10.3
Levothyroxine supplementation prior to hospital stay (number of cases/percentage* [%])	15/8.6
History of hyperthyroidism (number of cases/percentage* [%])	2/1.1
Thyrostatic use prior to hospital stay (number of cases/percentage* [%])	2/1.1
History of arterial hypertension (number of cases/percentage* [%])	102/58.6
History of diabetes (number of cases/percentage* [%])	53/30.5
History of asthma and/or chronic obstructive pulmonary disease (number of cases/percentage* [%])	18/10.3
History of chronic kidney disease (number of cases/percentage* [%])	26/14.9
History of hemodialysis (number of cases/percentage* [%])	17/9.8
History of heart failure (number of cases/percentage* [%])	17/9.8
History of atrial fibrillation(number of cases/percentage* [%])	23/13.2
History of stroke (number of cases/percentage* [%])	11/6.3
History of pulmonary embolism (number of cases/percentage* [%])	3/1.7
History of active neoplasia (number of cases/percentage* [%])	14/8.1

All numbers were mathematically rounded to the first decimal place. *Related to patients with available information.

### Statistical analysis

Continuous data are presented as mean values and standard deviation (SD). Categorical data are presented as percentages. The normal distribution was verified by the Kolmogorov-Smirnov test. Continuous data from two groups were compared using the Student’s t test or the Mann Whitney U test, depending on the distribution. More than two groups were compared using the Cochrane Q test for reliable variables. Categorical data were compared using the Chi-square test and Fisher’s exact test. Correlations, depending on the distribution of variables, were evaluated using the Pearson correlation test or the Spearman correlation test. A P value less than 0.05 was considered statistically significant. Data were analyzed using the SPSS software v.21 (IBM, USA).

## Results

The final analysis included 174 COVID-19 positive individuals, of whom 160 had TG assessed at the baseline, 147 at the second time point of observation, and 102 at the third time point of observation. The baseline characteristics of the studied population are shown in **Table 1**. 12 of 174 (6,9%) patients had positive anti-TG abs. According to our analysis of the entire studied population, TG concentration decreased over time (p<0.001; p1,2 = 0.025, p1,3 = 0.001, p2,3 = 0.003), and a similar pattern was observed in GCG (p<0.001; p1,2=<0.001; p1,3=<0.001; p2,3=<0.001), but not in NGCG (p=0.806) (**Table 2**). The number and percentage of cases with an increased TG concentration (>77 ng/ml) is displayed in **Table 2**. Upon subanalysis of GCG and NGCG, we found that TG concentration was significantly lower in GCG at the second and third time points (p=0.033 and p=0.001, consecutively). There were no statistically significant differences for the first time point (p=0.787) (**Table 3**). 

**Table 2:** TG concentration and the number of cases that exceeded the upper normal limit of TG concentration at three observation points in the general population and those treated versus those not treated with glucocorticoids.

	Time point of observation			
	1	2	3	
Group	General n=174			p value
TG (ng/ml), mean±SD	38.3 ± 55.0	32.2 ± 45.2	26.6 ± 41.3	<0.001* p1,2=0.025 p1,3=0.001 p2,3=0.003
TG>N, number of cases (%)	15 (8.6)	13 (7.5)	5 (3.0)	0.174*
Subgroup	GCG n=53			X
TG (ng/ml), mean±SD	31.3 ± 45.2	26.9 ± 46.3	21.7 ± 46.2	<0.001* p1,2<0.001 p1,3<0.001 p2,3<0.001
TG>N, number of cases (%)	7 (13.2)	7 (13.2)	2 (3.8)	0.379*
Subgroup	NGCG n=33			X
TG (ng/ml), mean±SD	44.3 ± 65.6	40.3 ± 54.7	34.2 ± 38.0	0.806*
TG>N, number of cases (%)	8 (24.2)	6 (18.2)	3 (9.1)	0.804*

GCG, patients treated with glucocorticoids; NGCG, patients not treated with glucocorticoids; N, upper limit of the norm. The General group: the mean TG concentration and the number of cases exceeding N were calculated for the entire population. The GCG and NGCG subgroups: the mean TG concentration and the number of cases exceeding N were calculated for patients with relevant data available for all three observation points.

*The marked p values were calculated for patients with relevant data available for all three observation points (General: n=86; GCG: n=53; NGCG: n=33).

Other p values were calculated for patients with relevant data available, with time points compared as indicated by numbers.

At the same time, TG concentration did not differ between the patients with normal and abnormal TFTs at any of the predesigned follow-up time points (**Table 3**). Abnormal TFTs were defined as laboratory results that deviated from the standardized norms provided by the manufacturer, and the parameters evaluated included TSH, T4, fT4, fT3, and rT3.

**Table 3:** TG concentration and cases with TG exceeding the upper limit of the norm: A. in individuals with abnormal versus normal results of thyroid function tests; B. in individuals treated versus not treated with glucocorticoids.

		Time point of observation								
		1			2			3		
**A**	Parameter	Abnormal TFTs n=98	Normal TFTs n=62	p	Abnormal TFTs n=98	Normal TFTs n=48	p	Abnormal TFTs n=79	Normal TFTs n=23	p
	TG (ng/ml), mean±SD	42.6 ± 66.7	31.5 ± 26.9	0.365	32.3 ± 44.9	32.5 ± 46.4	0.305	26.2 ± 45.4	27.9 ± 23.2	0.864
	TG>N, number of cases (%)	11 (11.2)	4 (6.5)	0.313	10 (10.2)	3 (6.1)	0.545	4 (5.1)	1 (4.3)	1.000
				
**B**		GCG n=53	NGCG n=107	p	GCG n=68	NGCG n=77	p	GCG n=59	NGCG n=39	p
	TG (ng/ml), mean±SD	38.1 ± 55.6	38.4 ± 55.0	0.787	25.2 ± 32.3	38.2 ± 53.8	0.033	21.3 ± 44.6	34.9 ± 37.2	0.001
	TG>N, number of cases (%)	5 (9.4)	10 (9.3)	1.000	5 (7.2)	8 (10.4)	0.506	2 (3.4)	3 (7.7)	0.384

TFTs, thyroid function tests; GCG, patients treated with glucocorticoids; NGCG, patients not treated with glucocorticoids; N, upper limit of the norm.

Values were calculated for patients with relevant data available.

Next, we wanted to verify the possible correlations between the concentration of TG, the parameters of thyroid function, and the inflammatory parameters. We did not find statistically significant correlations within the GCG and NGCG (**Table 4**). Statistical significance was found only after the analysis of the entire population studied at the third time point and included a positive correlation between TG and fT3 (r=0.215, p=0.030), TG and T4 (r=0.196, p=0.048), and TG and CRP (r=0.225, p=0.027).

**Table 4:** Relationship between TG concentration and various inflammatory and hormonal parameters.

Group	General n=174					
Parameters	Time point					
	1		2		3	
	r	p	r	p	r	p
TG-TSH	-0.149	0.061	0.097	0.245	0.082	0.413
TG-fT3	-0.002	0.982	0.022	0.792	0.215	0.030
TG-fT4	0.076	0.336	-0.068	0.412	0.029	0.771
TG-T4	0.010	0.903	-0.050	0.551	0.196	0.048
TG-LEU	-0.065	0.411	-0.054	0.515	-0.175	0.078
TG-NEU	-0.068	0.393	-0.094	0.263	-0.172	0.083
TG-LYMPH	-0.099	0.214	0.094	0.261	-0.009	0.930
TG-CRP	0.054	0.500	0.053	0.524	0.225	0.027
TG-IL-6	0.019	0.817	0.082	0.341	0.101	0.334
Subgroup	GCG					
Parameters	Time point					
	1		2		3	
	r	p	r	p	r	p
TG-TSH	-0.146	0.296	0.116	0.346	0.061	0.644
TG-fT3	0.250	0.071	0.123	0.319	0.076	0.568
TG-fT4	0.158	0.258	0.005	0.965	0.026	0.843
TG-T4	0.191	0.176	-0.060	0.625	0.093	0.482
TG-LEU	-0.270	0.050	-0.049	0.694	0.040	0.765
TG-NEU	-0.270	0.050	-0.100	0.421	0.048	0.719
TG-LYMPH	0.148	0.289	0.113	0.362	-0.010	0.940
TG-CRP	-0.002	0.987	0.114	0.357	0.250	0.061
TG-IL-6	-0.120	0.397	0.138	0.281	0.094	0.512
Subgroup	NGCG					
Parameters	Time point					
	1		2		3	
	r	p	r	p	r	p
TG-TSH	-0.128	0.193	0.007	0.955	-0.104	0.533
TG-fT3	-0.092	0.348	-0.200	0.083	0.120	0.468
TG-fT4	0.039	0.687	-0.140	0.226	0.169	0.304
TG-T4	-0.070	0.472	-0.052	0.654	0.135	0.413
TG-LEU	0.038	0.694	-0.001	0.991	-0.270	0.097
TG-NEU	0.021	0.831	-0.012	0.918	-0.205	0.210
TG-LYMPH	-0.176	0.070	-0.044	0.705	-0.131	0.428
TG-CRP	0.063	0.516	0.008	0.944	-0.070	0.684
TG-IL-6	0.091	0.358	0.022	0.856	-0.121	0.469

Values were calculated for patients with relevant data available. GCG, patients treated with glucocorticoids; NGCG, patients not treated with glucocorticoids.

To provide further insight, we analyzed the 19 cases of patients who had elevated TG levels at various points in the observation period (**Table 5**). Interestingly, only one patient had a TFT pattern suggestive of thyrotoxicosis with undetectably low TSH and increased fT4 (case #7), and one additional patient showed only a slight decrease in TSH with elevated fT4 (case #15). Two additional patients presented with a decrease in TSH without an increase in fT3 or fT4 (cases #6 and #16), and one of them had a known history of hyperthyroidism. The rest of the patients who showed elevated TG concentrations had TSH, fT3, and fT4 not indicative of thyrotoxicosis.

**Table 5:** Summary of patients who showed elevated TG.

Case number	Age (years)	Sex	History of hypothyroidism	History of hyperthyroidism	Positive anti-TG abs	Time point of observation	Steroid use prior to blood collection	TSH (μIU/ml; N: 0.27-4.2)	fT3 (pmol/l; N: 3.1-6.8)	fT4 (pmol/l; N: 12-22)	TG (ng/ml; N: 3.5-77)
1.	71	F	No	No	No	#2	Yes	2.82	1.78	15.17	88.5
2.	94	M	No	No	No	#1	No	2.15	2.23	11.1	117
						#2		1.78	2.1	11.56	88
3.	83	F	No	No	No	#1	No	0.493	2.92	14.46	205
						#2		0.635	3.11	12.97	138
						#3		1.37	3.32	14.4	103
4.	83	M	Yes	No	No	#1	No	8.55	2.5	14.65	296
						#2		17.42	2.73	15.78	311
						#3	Yes	6.24	1.96	14.37	332
5.	57	F	Yes	No	No	#1	No	1.03	4.2	16.54	97.5
						#2		1.79	3.96	14.84	80
6.	65	F	No	Yes	No	#1	Yes	0.29	3.15	13.62	158
						#2		0.215	3.32	15.08	103
7.	73	M	No	No	No	#1	No	<0.005	3.18	23.66	133
						#2		<0.005	2.63	15.76	165
8.	71	F	Yes	No	No	#1	Yes	0.827	1.58	15.73	194
						#2		0.897	2.25	17.17	165
9.	70	F	No	No	No	#2	No	4.62	3.78	15.48	88.3
						#3		4.92	4.14	17.99	91.2
10.	80	M	No	No	No	#1	No	0.719	3.17	15.61	94.4
11.	73	M	No	No	No	#1	Yes	3.37	3.76	15.12	81.3
						#2		1.18	2.56	13.57	139
12.	69	F	No	No	No	#1	No	0.787	1.8	15.33	87.5
13.	59	M	No	No	No	#1	No	1.66	6.1	16.03	92.4
14.	61	F	No	No	No	#1	No	0.851	2.4	10.72	271
15.	85	F	No	No	No data	#3	Yes	0.24	3.14	25.38	77.9
16.	72	M	No	No	No	#1	Yes	0.145	1.85	12.05	333
17.	82	F	No	No	No	#2	No	0.793	3.08	11.44	120
18.	86	F	No	No	No	#1	Yes	1.19	3.06	12.25	131
						#2		1.31	2.61	16.35	132
19.	90	F	No	No	No	#1	No	1.28	3.04	16.96	346
						#2		2.29	3.15	18.04	304
						#3		2.52	3.05	19.12	207

F, female; M, male; N, normal value.

Finally, we wanted to verify whether the TG concentrations differed between patients who met and those who did not meet the predefined endpoints. The combined primary endpoint consisted of death, mechanical ventilation, noninvasive ventilation/high flow nasal oxygenation, use of vasopressors, hospitalization for at least 10 days; the secondary endpoints were any of the listed events. First, we analyzed the entire study population: there were no statistically significant differences between patients who met and those who did not meet the combined primary endpoint, both in terms of TG concentration and the incidence of high TG concentration. No differences between groups were found at any of the follow-up points for secondary endpoints, such as prolonged hospital stay, mechanical ventilation, and vasopressor infusion. Statistically significant results were found in relation to death (the first time point [p=0.031] and the second time point [p=0.005], but not the third time point [p=0.091]), and noninvasive ventilation (only the second time point with p=0.043). GCG and NGCG were not evaluated separately.

## Discussion

The relationship between COVID-19 and thyroid function quickly gained widespread coverage from medical researchers. The range of topics evaluated was broad and included NTI, SAT, autoimmune thyroiditis, and a possible link between TFTs and survival in COVID-19 (1, 3, 15–18). The involvement of the gland appeared to be multifactorial, resulting both from the direct effects of viral infiltration and the local response it provoked, as well as systemic inflammation, often exaggerated up to the phase of the cytokine storm (1, 4, 19). The cytokine storm, with its uncontrolled and robust release of pro-inflammatory cytokines, such as interleukin-6 (IL-6), interleukin-1 (IL-1), and tumor necrosis factor-alpha (TNF-α), is the peak of the detrimental immune hyperactivation seen in COVID-19 (20–22). SARS-CoV-2 penetrates cells using ACE2 receptors, which are widespread throughout the human system, and viral entry is co-facilitated by the serine protease TMPRSS2 (23–26). Therefore, the virus can infiltrate numerous tissues and organs, including thyroid (19, 24).

A systematic review of 1,237 cases of COVID-19 from 7 selected studies carried out by Giovanella et al. proved that abnormal thyroid function in COVID-19 is common and up to 64% of patients display abnormal TFTs (3). Our recent study on thyroid function in patients hospitalized for COVID-19 supported these findings: up to 80% of recruited individuals showed abnormal TFTs at least once during the observation period (27). In particular, NTI appeared to be common in patients with COVID-19 (27, 28). NTI features tended to fade over time in patients recovering from COVID-19, but remained prominent in patients who succumbed to their illness (29). A positive correlation was observed between abnormal TFTs and high inflammatory markers, and abnormal TFTs could herald an unfavorable clinical course of the disease and a prolonged hospital stay (27, 30).

The results of the THYRCOV study by Lania et al. proved that thyrotoxicosis might not be rare during COVID-19, as it was found in more than 20% of the studied patients. At the same time, thyrotoxicosis was positively correlated with elevated circulating IL-6 (14). On the other hand, a study by Campi et al. showed a considerable prevalence of transiently decreased TSH, often accompanied by low fT3. In this group, TG levels remained normal despite the decrease in TSH at baseline or after follow-up (29). The authors concluded that the pattern of observed TFT abnormalities was most likely not related to destructive thyroiditis, and rare instances of elevated TG could be associated with a known preexisting condition (nodular goiter) (29). In another study, Vassiliadi et al. reported that TG levels were comparable between COVID-19 patients with normal TFTs, NTI, or a thyrotoxic pattern. The thyrotoxic pattern was observed in 8.8% of all patients who suffered from COVID-19, and it was seen in 14.6% of severely ill individuals who needed intensive care unit treatment (28).

An observational study by Mondal et al. showed that the prevalence of SAT in COVID-19 convalescents was 6.8% (n=11) within 3 months of follow-up - most of the patients were young (mean age 44 years) and female, the mean delay between COVID-19 and the diagnosis of SAT was 23,8 days, and the clinical course of the disease was more severe in atypical SAT, which presented earlier (10,6 days since the recovery from COVID-19), with an exaggerated inflammatory response and more pronounced thyrotoxicosis (12). SAT became an interest in endocrine research after Brancatella et al. presented the first known case report of COVID-19-related SAT in a young woman in mid-2020 (6). There is evidence that certain populations are more at risk of developing SAT due to their specific human leukocyte antigen (HLA) system, COVID-19-related SAT included (7, 8, 31, 32). The first stage of thyroiditis, thyrocyte breakdown resulting in hyperthyroidism, is marked not only by an uninhibited leak of peripheral thyroid hormones from the damaged gland, but also by a simultaneous release of TG, a glycoprotein stored within the thyroid follicular lumen, crucial for hormonogenesis and considered a biomarker of destructive thyroiditis (29, 33). In our project, we wanted to assess markers of possible thyroid destruction during COVID-19. Increased TG concentration in the bloodstream is not limited to SAT; it can reflect the amount of differentiated thyroid tissue, the degree of stimulation of the gland related to thyrotropin, and the history of recent thyroid trauma (33, 34). Therefore, TG concentration can increase in various clinical settings, including SAT, differentiated thyroid cancer, nodular goiter, or hyperthyroidism. As mentioned above, a pattern of TFTs suggesting thyrotoxicosis in patients who showed elevated TG concentration was not a common finding in our study. In general, we did not find clear, repeatable, and unequivocal relationships between TG levels, TFTs, and inflammatory parameters, which may suggest an overall limited destructive effect of COVID-19 on thyroid tissue. Interestingly, TG levels dropped after the introduction of GCs, a phenomenon previously described by researchers who focused on the management of SAT, which could be explained, among others, by impaired intrathyroidal hydrolysis of colloid due to the use of GC or a decline in systemic inflammation (35–37).

The main limitations of this study include the lack of post-discharge follow-up, especially since the available data highlight the frequent delay between COVID-19 and SAT; no in-hospital assessment specific for clinical signs and symptoms of SAT; the absence of radiological evaluation of the thyroid in terms of SAT or other pathologies that cause increased TG release, such as goiter. A combination of the aforementioned factors and additional laboratory work could give better insight into the characteristics necessary to adequately document the occurrence of SAT. However, the assessment we designed was not meant to unequivocally determine the number of SAT cases in the studied population, but to investigate possible generalized thyroid damage based on TG levels, TFTs, and their correlation with inflammatory markers. Listing the limitations of the study, we must mention that a small percentage of the recruited patients (12 out of 174 – 6,9%) had positive anti-TG abs, which can sometimes interfere with the evaluation of TG. In addition, some of the presented statistical analyses, especially related to the entire studied population, might be biased due to inconsistent study samples (e.g. exogenous steroid use in some patients – GCG) and, sometimes, a wide disproportion between the number of studied cases.

The strengths of our study are the prolonged period of in-hospital observation with three separate assessment points, the subanalyses based on the use of GCs or its lack, the consistent set of performed assays, the in-depth analysis of detected cases with increased TG, and the assessment of TG levels in relation to predefined endpoints. We believe that, despite its limitations, our study still sheds new light on the complicated matter of TFTs and thyroid function abnormalities in COVID-19.

### Summary

The thyroid function in COVID-19 is complex and sometimes confusing. COVID-19-related NTI, SAT, and thyrotoxicosis quickly gained the attention of researchers. TFTs undergo distinct and very often transient changes in COVID-19-positive patients. In our material, the concentration of TG did not differ between the patients with normal and abnormal TFTs; however, it differed significantly depending on the use of GCs and tended to decrease over time. We found that 19 patients had elevated TG levels at least once during observation, but a TFT pattern suggestive of thyrotoxicosis was not common in this group. There were no statistically significant differences between patients who met and those who did not meet the combined primary endpoint.

## Data availability statement

The raw data supporting the conclusions of this article will be made available by the authors upon reasonable request.

## Ethics statement

The studies involving human participants were reviewed and approved by the Independent Bioethics Committee for Scientific Research at the Medical University of Gdańsk. Patients/participants provided their written informed consent to participate in this study. The Independent Bioethics Committee for Scientific Research at the Medical University of Gdańsk waived the requirement of written consent in patients unable to provide it due to the bad general state caused by COVID-19 and/or concomitant diseases.

## Author contributions

RŚ-S – manuscript concept and preparation, results interpretation, manuscript revision, literature collection and review, project supervision. AB – manuscript concept and preparation, database creation, results interpretation, literature collection and review. EP-R – patient recruitment, data collection. All authors contributed to the article and approved the submitted version.
