# Supplementary material for: Thyroglobulin levels in COVID-19-positive patients: Correlations with thyroid function tests, inflammatory markers, and glucocorticoid use
Source: Front Endocrinol (Lausanne). 2023 Mar 9;13:1031188. doi: 10.3389/fendo.2022.1031188 (PMC10034180; doi:10.3389/fendo.2022.1031188)
Supplement: Supplementary file 1 [file Table_1.pdf]

Supplementary Table: Detailed information on used laboratory assays.

	Parameter	Reference norm with units	Laboratory	Analytical method	Laboratory kit
1.	TSH	0.27-4.2 µIU/ml	Diagnostyka Laboratoria, Gdańsk, Poland	Electrochemiluminescence assay	Elecsys TSH, Roche Diagnostics, Germany, Ref. 08429324190
2.	fT4	12-22 pmol/l	Diagnostyka Laboratoria, Gdańsk, Poland	Electrochemiluminescence assay	Elecsys FT4 III, Roche Diagnostics, Germany, Ref. 07976836190
3.	fT3	3.1-6.8 pmol/l	Diagnostyka Laboratoria, Gdańsk, Poland	Electrochemiluminescence assay	Elecsys FT3 III, Roche Diagnostics, Germany, Ref. 06437206190
4.	T4	5.13-14.10 µg/dl	Diagnostyka Laboratoria, Gdańsk, Poland	Electrochemiluminescence assay	Elecsys T4, Roche Diagnostics, Germany, Ref. 09007784190
5.	TG	3.5-77 ng/ml	Diagnostyka Laboratoria, Gdańsk, Poland	Electrochemiluminescence assay	Elecsys Tg II, Roche Diagnostics, Germany, Ref. 07027931190
6.	rT3	i. <0.95 ng/ml<br>ii. 0.1-0.35 µg/l	i. Central Clinical Laboratory, University Clinical Centre, Gdańsk, Poland<br>ii. Cerba International, Barcelona, Spain	i. Chemiluminescence immunoassay<br>ii. Radioimmunoassay	i. Maglumi Rev T3 (CLIA), Snibe Diagnostic, China, Ref. 130203010M<br>ii. Reverse T3-RIA, DiaSource ImmunoAssays, Belgium, Ref. R-EW-125
7.	anti-TG abs	<10 IU/ml	Diagnostyka Laboratoria, Gdańsk, Poland	Electrochemiluminescence assay	Elecsys Anti-TG, Roche Diagnostics, Germany, Ref. 07026919190
8.	CRP	0-5 mg/l	Diagnostyka Laboratoria, Gdańsk, Poland	Immunoturbidimetric assay	Tina-quant C-Reactive Protein IV, Roche Diagnostics, Germany, Ref. 07876033190
9.	IL-6	<5.9 pg/ml	Central Clinical Laboratory, University Clinical Centre, Gdańsk, Poland	Chemiluminescence immunoassay	Immulite 2000 IL-6, Siemens Healthcare, Germany, Ref. L2K6P2
10.	LEU	females: 3.98-10.04x10^{3}/µl<br>males: 4.23-9.07x10^{3}/µl	Diagnostyka Laboratoria, Gdańsk, Poland	Fluorescence flow cytometry	Stromatolyser 4DS, Sysmex Corporation, Japan, Ref. 98417216; Stromatolyser-FB, Sysmex Corporation, Japan, Ref. 94404613; Stromatolyser 4DL, Sysmex Corporation, Japan, Ref. 98417615; Sulfolyser, Sysmex Corporation, Japan, Ref. 90411414
11.	NEU	2-7x10^{3}/µl	Diagnostyka Laboratoria, Gdańsk, Poland		
12.	LYMPH	1-3x10^{3}/µl	Diagnostyka Laboratoria, Gdańsk, Poland		
